# Effects of X-Rays, Electron Beam, and Gamma Irradiation on Chemical and Physical Properties of EVA Multilayer Films

**DOI:** 10.3389/fchem.2022.888285

**Published:** 2022-05-11

**Authors:** Nina Girard-Perier, Sylvain R. A. Marque, Nathalie Dupuy, Magalie Claeys-Bruno, Fanny Gaston, Samuel Dorey, Leonard S. Fifield, Yelin Ni, Donghui Li, Witold K. Fuchs, Mark K. Murphy, Suresh D. Pillai, Matt Pharr, Larry Nichols

**Affiliations:** ^1^ Sartorius Stedim FMT S. A. S., Aubagne, France; ^2^ Case 551, CNRS, ICR, Aix Marseille University, Marseille, France; ^3^ CNRS, IRD, IMBE, Aix Marseille University, Avignon Université, Marseille, France; ^4^ Pacific Northwest National Laboratory, Richland, WA, United States; ^5^ National Center for Electron Beam Research, Texas A&M University, College Station, TX, United States; ^6^ Department of Mechanical Engineering, Texas A&M University, College Station, TX, United States; ^7^ Steri-Tek, Fremont, CA, United States

**Keywords:** methionine oxidation, electron spin resonance, liquid chromatography, differential scanning calorimetry, gamma, x-ray, e-beam irradiation

## Abstract

Gamma-ray irradiation, using the cobalt-60 isotope, is the most common radiation modality used for medical device and biopharmaceutical products sterilization. Although X-ray and electron-beam (e-beam) sterilization technologies are mature and have been in use for decades, impediments remain to switching to these sterilization modalities because of lack of data on the resulting radiation effects for the associated polymers, as well as a lack of education for manufacturers and regulators on the viability of these sterilization alternatives. For this study, the compatibility of ethylene vinyl acetate (EVA) multilayer films with different ionizing radiation sterilization (X-ray, e-beam, and gamma irradiation) is determined by measuring chemical and physical film properties using high performance liquid chromatography, differential scanning calorimetry, Fourier-Transform InfraRed spectroscopy (FTIR), surface energy measurement, and electron spin resonance techniques. The results indicate that the three irradiation modalities induce no differences in thermal properties in the investigated dose range. Gamma and X-Ray irradiations generate the same level of reactive species in the EVA multilayer film, whereas e-beam generates a reduced quantity of reactive species.

## 1 Introduction

A major use of ionizing radiation is for irradiation of polymer-based products that require sterilization. Currently, gamma radiation, provided by the cobalt-60 isotope, is the most common radiation modality used for medical device and biopharmaceutical product sterilization. The two alternative irradiation modalities, electron beam (referred to as e-beam) and X-ray, although mature and effective sterilization technologies, have remained a small percentage of the sterilization market. All types of ionizing radiation can modify physical-mechanical-tribological and chemical properties of polymers. Most of the scientific publications that report the beneficial and deleterious impacts of radiation on polymers involve gamma radiation (Montanari et al., 1998; Kang and Nho, 2001; Buttafava et al., 2005; Abdel Tawab et al., 2013) and publicly available data on polymer effects from e-beam and X-ray radiation (Croonenborghs et al., 2007; Fifield et al., 2021) are lacking for many types of polymers, including those involved with multilayer films for biopharmaceutical applications. One prior article reports on the effect of high energy X-ray and gamma radiation on the mechanical properties of different polymers (Croonenborghs et al., 2007), and both radiation types display very similar effects. There are limited works in the published literature reporting investigation of the effects of X-ray radiation on polymers (Takahagi et al., 1990; Graham et al., 1997; Coffey et al., 2002; Manfredini et al., 2003; Ng and Yu, 2006). A few articles have compared the effects of gamma irradiation and e-beam irradiation on mechanical properties of polypropylene. Fintzou et al. (2007) highlighted that gamma irradiation of polypropylene had a greater effect on mechanical and thermal properties than did e-beam irradiation (i.e., decrease in load, elongation at break, and lower decrease in melting temperature for gamma irradiated samples). Hassan et al. (2008) showed that the degradation of highly crystalline polypropylene properties caused by gamma irradiation was higher than that caused by e-beam irradiation. Finally, Badia et al.(Badia and Duplâtre, 1999) compared the effects of gamma irradiation and e-beam irradiation on high density polyethylene, and they found no change in the shelf life.

In this present study, the impacts of three irradiation modalities have been investigated: X-ray, e-beam, and cobalt-60 gamma. EVA single use bags made of a multilayer film composed of ethylene vinyl acetate (EVA)/ethylene vinyl alcohol (EVOH)/EVA are currently and commonly used in biopharmaceutical field and have been chosen as a model. Recently, the effects of gamma irradiation on the physical/mechanical/chemical properties of these multilayer films were thoroughly studied using eight different techniques (Gaston et al., 2016, 2017, 2018; Dorey et al., 2018a; Dorey et al., 2018b; Girard-Perier et al., 2020b; Dorey et al., 2020), with an established worse-case irradiation dose set at 50 kGy for gamma-rays to exceed the routine irradiation dose range (i.e., approximately 25–45 kGy). In that recent study, the X-ray irradiation dose was set at approximately 20 kGy above this gamma-ray established worse-case dose to explore its effect on plastics. This present study investigates polymer modification under these three irradiation modalities, as to provide insight into general multilayer structure attributes for use in biopharmaceutical applications, or in interaction with biopharmaceutical solutions. The changes in transition temperatures and heat capacity (Dorey et al., 2020) were determined by differential scanning calorimetry (DSC) as a marker of material degradation. The generation of reactive species such as peroxides and peracids, due to radical post reactivity, were probed by the methionine oxidation (mimicking the oxidation of proteins) monitored by high performance liquid chromatography (HPLC). Long-lived radicals were tracked by electron spin resonance (ESR).

## 2 Materials and Methods

### 2.1 Sample Bags

**FIGURE 1:**
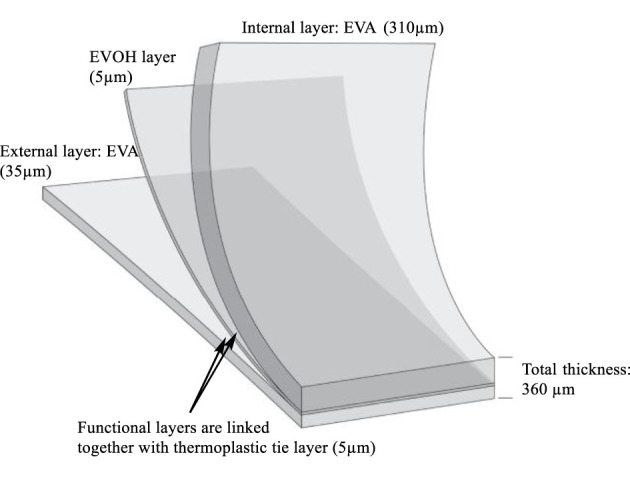
Structure of the EVA/EVOH/EVA multilayer film.

The single-use plastic bags studied were made from an EVA-based multilayer film composed of one layer of EVOH (5 µm) sandwiched between two layers of EVA, with a total thickness of about 360 µm (Figure 1). Polyethylene (PE) was present as a tie layer (5 µm) between the EVA and EVOH.

### 2.2 Gamma Irradiation

EVA bags (series #1) and pre-cut EVA films (series #2) were packed and wrapped in multilayer packaging (polyethylene/polyamide/polyethylene) of thickness 100 ± 20 µm and irradiated at room temperature with a cobalt-60 gamma source at Ionisos, Dagneux, France. Irradiation was performed under environmental atmosphere. For series #1, the average target dose was 59 ± 3 kGy for the methionine oxidation evaluation, 54.3 ± 2.7 kGy for the thermal property evaluation, and 48.2 ± 2.1 kGy for the surface energy evaluation. For series #2, the average measured gamma doses for samples used in methionine oxidations, thermal property and surface energy evaluation were 30.0 ± 1.9 kGy, 44.6 ± 0.3 kGy, and 60.9 ± 1.1 kGy. The dose rate provided was of 1–2 kGy/h. Alanine dosimeters were used on the cardboard box containing the samples to assess the radiation delivered to the single use bag samples (±5%). To obtain the target dose, it was necessary to perform several sterilization cycles, including a waiting time not controlled between each cycle. All the boxes have been exposed to a double-sided irradiation.

### 2.3 E-Beam Irradiation

EVA bags (series #1) were individually wrapped in multilayer packaging (polyethylene/polyamide/polyethylene) and placed side-by-side in a thin cardboard box (8 cm) to have only one thickness of plastic material. Bags (series #1) were irradiated with a 10 MeV Rhodotron (Ionisos, Chaumesnil, France) at a dose rate of 18,000 kGy/h with a power source at 28 kW. Alanine dosimeters were used on cardboard boxes containing the samples to assess the radiation delivered to single use bag samples (±5%). The range in delivered surface dosage was 51 ± 1 kGy using double-sided irradiation. These samples were used for thermal property, surface energy, and methionine oxidation evaluation.

Pre-cut films (series #2) were processed using two 10 MeV, 20 kW e-beam Mevex accelerators at Steri-Tek (steri-tek.com) in Fremont, California, CA, United States B3 dosimeters were used on cardboard boxes containing the samples to assess the radiation delivered to film samples used in thermal property, surface energy, and spectroscopic evaluation to be delivered at 31 ± 2, 47 ± 2, and 59 ± 3 kGy.

### 2.4 X-Ray Irradiation

EVA bags (series #1) were individually wrapped in multilayer packaging (polyethylene/polyamide/polyethylene). They were irradiated with a 7 MeV Rhodotron (Steris, Däniken Switzerland) with an average dose rate of 50 ± 30 kGy/h with a maximum power source of 560 kW. The range in delivered surface dosage was 68 ± 0.6 kGy for the methionine oxidation evaluation using double-sided irradiation. EVA bags (series #1) used in other tests were irradiated with a 7 MeV Rhodotron (Aerial, Strasbourg France). The range in delivered surface dosage was 50.6 ± 4.0 kGy for the thermal property measurements, and 54.7 ± 2.5 for the surface energy evaluation using double-sided irradiation.

EVA film samples (series #2) were wrapped in multilayer packaging (polyethylene/polyamide/polyethylene). They were irradiated with a 7 MeV Rhodotron (Aerial, Strasbourg France) with an average dose rate of 15 kGy/h. The delivered surface dosages were 33.2 ± 0.4 kGy, 45.3 ± 0.6, and 59.8 ± 1.1 kGy using double-sided irradiation. An EVA bag irradiated up to 98.9 ± 0.7 kGy was also included in series #2. Series #2 samples were used in thermal property, surface energy, and spectroscopic evaluation.

### 2.5 High Performance Liquid Chromatography

Three months after irradiation, bags were filled with a 50 µM solution of methionine in buffer (10 mM NaH_2_PO_4_, 10 mM Na_2_B_4_O_7_•10H_2_O, 5 mM NaN_3_, pH 8.2). After storage for 10 days sampling was performed and the solution analyzed with an Agilent 1260 HPLC equipped with a quaternary pump (G1311C), an autosampler (G1329B), and a fluorescence detector (G1321B). Separation between the methionine and its sulfoxide form was carried out on an Agilent Poroshell HPH-C18 column (4.6 mm × 100 mm, 2.7 μm particles) used with a UHPLC guard Poroshell HPH-C18, 4.6 mm pre-column. Details of chemicals and reagents used, HPLC system and conditions are described in a previous article (Girard-Perier et al., 2020a).

### 2.6 Differential Scanning Calorimetry

The two series of samples were analyzed within 3 months after irradiation. The melting temperatures of series #1 and series #2 were determined using DSC. EVA bags (series #1) were measured on a Sensys Evo DSC (Setaram, Lyon, France) from 23 to 250°C at 10°C/min heating and cooling rates. Melting temperature was determined in second and third heating cycles for 2–3 samples per data point. More details were described in a previous article (Dorey et al., 2020). The pre-cut films (series #2) were tested on a TA Instruments (Waters LLC, New Castle, DE, United States ) Q2000 DSC. The melting temperatures were determined in the second heating cycle from −140 to 200°C at 10°C/min, after a 10°C/min cooling step from 200°C to −140°C. Three replicates of films (series #2) were tested at each irradiation condition. Unirradiated specimens in series #1 and #2 were measured on corresponding DSC instruments to calibrate systematic differences.

### 2.7 Electron Spin Resonance

The samples were analyzed within 10 days following irradiation. ESR measurements were carried out on a Bruker EMX X-band spectrometer operating at 9.5 GHz and equipped with a highly sensitive rectangular microwave cavity. The spectroscopic parameters were modulation amplitude 2 G, magnetic field sweep 500 G, receiver gain 103, resolution 1,024 points, power 20.12 mW, and sweep time of 20.972 s. Four scans were performed to record each ESR signal.

### 2.8 Surface Energy

Surface energy measurements were performed on EVA bags (series #1) using a GBX goniometer at controlled temperature and humidity (23°C/50% RH). Measurements were performed with ethylene glycol, purified water, and diiodomethane with respective capillary volume (2.097 µL; 2.72 µL; 1.24 µL). Ten droplets were deposited on each sample. The surface tension energies were calculated using the “Owens-Wendt-2 equation” from the average of the contact angle measurements for each liquid.

The surface energies were also measured on pre-cut films (series #2) using a Krüss Mobile Surface Analyzer (MSA) equipped with ADVANCE software and diiodomethane (Thermo Scientific; >99% purity) and distilled water syringes. Measurements were performed in quintuplicate using the double sessile drop program and an unmodified version of the automation program provided with the ADVANCE software. Droplet size (2 μL target) was calibrated every 10 measurements. Contact angles were measured using the automatic baseline function and the ellipse (tangent^−1^) fitting method. Erroneous contact angle measurements were corrected by the manual baseline method. The surface free energy was calculated using the Owens, Wendt, Rabel, and Kaelbe (OWRK) method with a correlation coefficient of 1.00. Additional parameters accounting for microscale surface roughness were not used in this work.

### 2.9 Fourier Transform Infrared

The surfaces of sample films (series #2) were characterized using a Bruker Alpha II OPUS Touch FTIR spectrometer with an attenuated total reflection (ATR) attachment. The specimens were conditioned following ASTM D618 Procedure A at 23 ± 2°C and 50 ± 10% relative humidity for at least 40 h. After conditioning, the spectra for each specimen were recorded from 4,000 to 700 cm^−1^. To reduce measurement variation, 64 scans were collected at a resolution at 4 cm^−1^ for each specimen, and each specimen was measured at four locations near four edges.

Peak absorbance intensities corresponding to the methylene group (maximum absorbance between 2,846 and 2,850 cm^−1^, CH_2_ symmetric stretch) and carbonyl group (1715 cm^−1^, C=O stretch) were used to calculate the carbonyl index. The carbonyl index (CI) is given in Eq. 1, where $A_{C=O}$ and $A_{C-H}$ are the absorbance peak values found in the ranges of 1,650–1830 cm^−1^ and 1,400–1,510 cm^−1^, respectively.

$$\text{Equation}1\text{:}CI\;=\frac{A_{C=O}}{A_{C-H}}$$ (1)

### 2.10 Storage Conditions

Samples were stored in boxes in the dark following irradiation, in an air-conditioned room at 20 ± 2°C.

## 3 Results and Discussion

**FIGURE 2:**
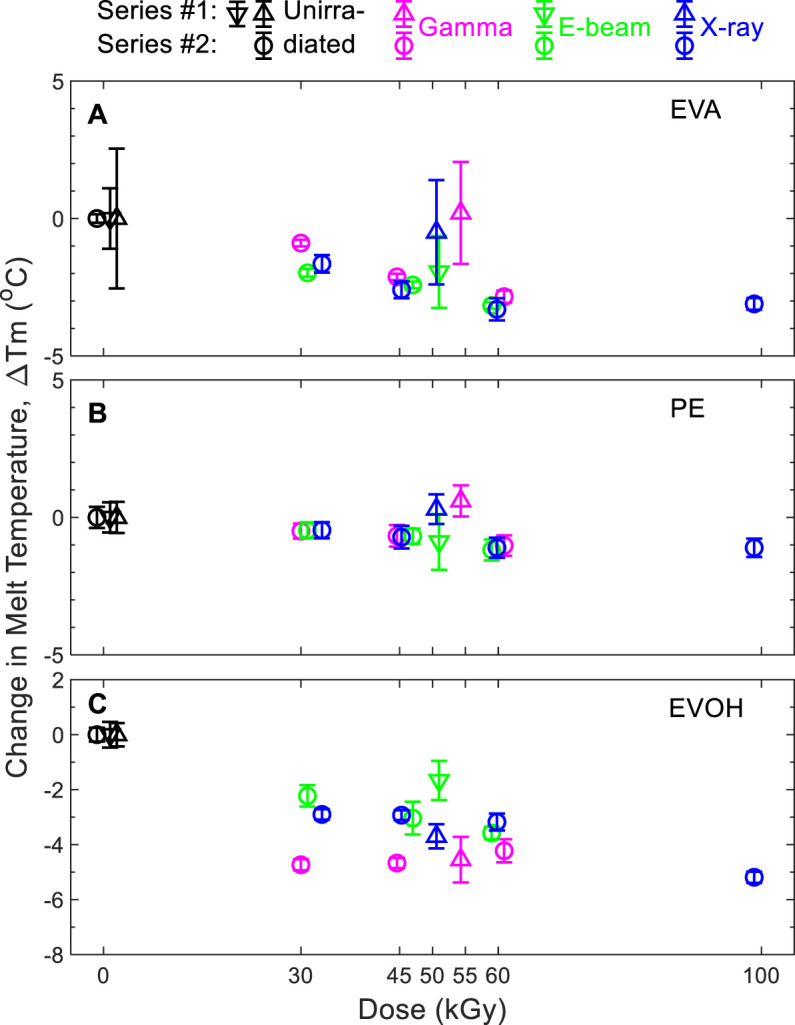
Change in melting temperatures of **(A)** EVA, **(B)** PE, and **(C)** EVOH components with respect to non-sterile films. Triangles: series #1. Circles: series #2. Triangles pointing up and down represent data obtained for samples from two different manufacturing batches. Representative DSC responses of EVA/EVOH/EVA films (series 2) are shown in Supplementary Figure S1.

ESR detection of radical species in this material after 3-months aging was unsuccessful both for e-beam and X-ray irradiation, which was in agreement with the absence of an ESR signal after gamma irradiation as previously reported (Audran et al., 2015). Therefore, neither e-beam nor X-ray irradiation generated more stable radical species than those expected under gamma irradiation. A previous study (Dorey et al., 2020) shows that there is no micro morphology difference between non irradiated and 50 kGy gamma irradiated samples. This feature was then not evaluated in this current study. The effects of gamma irradiation, e-beam, and X-ray on thermal characteristics of the EVA multilayer samples are shown in Figure 2 for the polyethylene (PE), EVA, and EVOH components. Data are displayed as the difference between the irradiated and unirradiated sample values to directly compare data measured by the partner organizations. Measurements of the melting points of EVA and EVOH layers showed close results between samples exposed to X-ray, e-beam, and those irradiated with a gamma source.

A statistical evaluation was achieved with an “equivalency test” using the software Minitab®. The equivalency criterion was 5°C. The Null hypotheses H_O_ for the mean values difference was either µ_Gamma_—µ_Xray_ ≤ −5°C or µ_Gamma_—µ_Xray_ ≥ 5°C. The alternative hypotheses H_1_ was either −5°C<µ_Gamma_—µ_Xray_ <0°C or 0°C<µ_Gamma_—µ_Xray_ < 5°C. The equivalency criterion used to check was whether the measurement results of gamma, X-ray, and e-beam irradiated samples fell within the equivalence interval. The statistical evaluation was an initial tool to check the clustering of data. The understanding of the polymer behavior remains the predominant parameter to finally evaluate the potential impact difference between all ionizing radiation modalities.

Results in Figure 2 demonstrate, whatever the irradiation modality, that the thermal properties for the EVA, PE, and EVOH layers are within the method uncertainties for the two series and can be considered as equivalent. Moreover, the subsequent statistical evaluation showed that the EVA is slightly impacted by all three sterilization modalities, with a tendency to decrease the melting temperature (T_m_) with increasing dose (up to 100 kGy) when compared to the non-treated (0 kGy) samples as the reference (Supplementary Table S1). The statistical evaluation showed that the PE is neither impacted by gamma nor X-ray nor e-beam up to 100 kGy (Supplementary Table S2). The statistical evaluation showed that the EVOH is not impacted by either gamma or X-ray or e-beam up to 60 kGy (Supplementary Table S3). The melting peak temperature values of the EVA-PE-EVOH materials irradiated by either gamma or X-ray or e-beam were distributed within the 5°C equivalence interval. There was no difference of the thermal properties of the EVA/EVOH/EVA film either irradiated by Gamma or X-ray or e-beam up to 60 kGy. Thermal transitions of the EVA layers that lend the film its mechanical robustness were observed to alter by less than 5°C with irradiation even up to 100 kGy. The impact on the EVOH layer at 100 kGy after gamma irradiation has previously been seen to primarily affect the oxygen barrier properties (Dorey et al., 2020).

**FIGURE 3:**
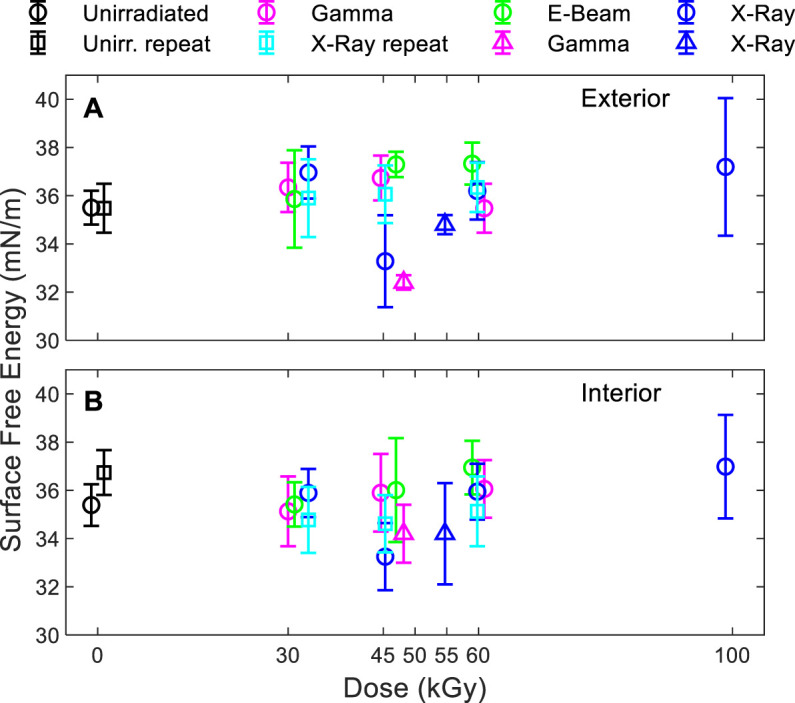
Surface free energy of the **(A)** exterior and **(B)** interior side of the bag. Triangles: series #1. Circles and squares: series #2.

While the effect of irradiation on the measured surface energy of the films was small (Figure 3), surface energy was observed to increase slightly with dose except for the 45 kGy X-ray and 48 kGy gamma scenarios. No trend in difference was observed between the results of the modalities.

**FIGURE 4:**
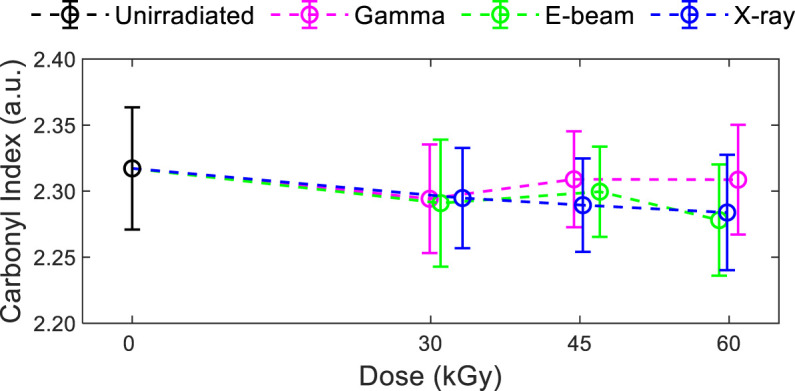
Carbonyl index of series #2 multilayer films. Representative FTIR response of EVA/EVOH/EVA films (series 2) are shown in Supplementary Figure S2.

Effects of dose and modality on carbonyl index showed a slight decrease with exposure as seen in Figure 4, although the values for exposed and unexposed samples were almost equivalent within the uncertainty of the measurements. The carbonyl index, the ratio of FTIR absorbance peak intensities for C=O and C=H bonds, tends to increase with oxidation for polyolefins. The slight decrease with exposure observed here indicates a lack of significant oxidation of the surface of the EVA multilayer films with irradiation up to 60 kGy. At the higher dose of 45 and 60 kGy, the carbonyl index values of the gamma irradiated samples are slightly larger than those for the other two modalities.

**FIGURE 5:**
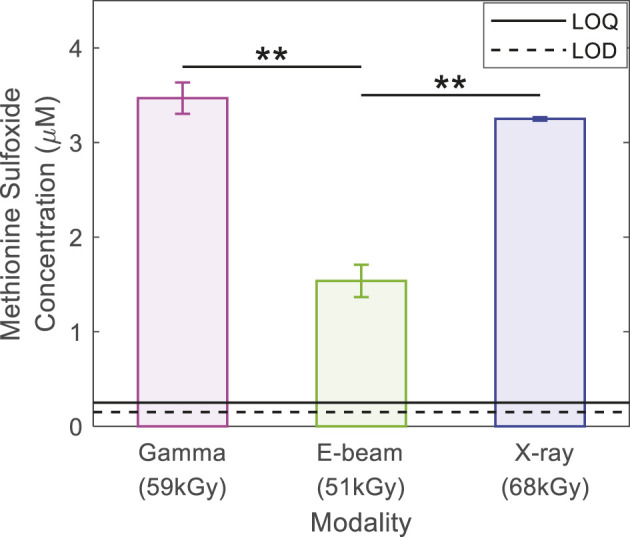
Methionine sulfoxide concentration (µM) in stored solution for 10 days in series #1 bags and analyzed by HPLC. Irradiation doses were 59 kGy for gamma irradiation, 51 kGy for e-beam and 68 kGy for X-rays. The solid line indicates the Limit of Quantification (LOQ), and the dashed line indicates the Limit of Detection (LOD). ** means that the difference is statistically significant, and *p*-value < 1%.

In contrast to DSC, surface energy and FTIR investigations, striking differences in the quantity of reactive oxygen species generated were observed with the irradiation modalities (Figure 5). In a previous article (Girard-Perier et al., 2020a), we explained how methionine is oxidized into methionine sulfoxide in gamma irradiated samples and we showed that, if no radical species were detected in the samples, oxidation was likely due to the presence of hydrogen peroxide, peracids, or *in situ* generated peracids. In the present study, the quantity of methionine sulfoxide formed subsequently after the gamma and X-ray irradiations was found to be equivalent (i.e., 3.5 ± 0.2 and 3.2 ± 0.2 µM respectively). These values were ∼two times more than that observed for e-beam irradiated samples, in Figure 5 (1.5 ± 0.2 µM). The difference observed of the methionine sulfoxide quantity between gamma and e-beam irradiated samples and the X-rays and e-beam irradiated samples was statistically significant (Student test) with a *p*-value < 1% (*p*-value = 0.008 and 0.005, respectively (Girard-Perier et al., 2020a)). The lower dosage (10 kGy less) in the e-beam irradiated samples cannot be the main root cause of the lower oxidation level after e-beam irradiation. These observations could mean that the process and the quantity of oxidizing species generated are the same with gamma rays and X-rays. The quantity of oxidizing species generated with e-beam and interacting with the polymer matrix could be altogether lower (Girard-Perier et al., 2021). It is widely described in literature that the interaction of high energy photons, such as gamma and X-ray, with matter leads to ionization through electron pair formation, the Compton effect (predominant), and photo elastic electron formation (Adliéné and Adlytė, 2017; Adliéné et al., 2017). In the first interaction step, the ionizing irradiation transfers energy to secondary charged particles (i.e., secondary electrons). The interaction of primary electrons also leads to the creation of secondary electrons. It is well known that e-beam irradiation is a faster process (GIPA and IIA, 2017) than gamma and X-ray irradiation due to its higher dose rate (Hassan et al., 2008). The space that the secondary electrons occupy is called a spur (Lee et al., 1999). When spurs overlap reactive species may interact together instead of reacting with the polymer matrix (Kozawa et al., 2009), potentially explaining the lower methionine sulfoxide quantities detected in e-beam irradiated samples.

## 4 Conclusion

EVA/EVOH-based multilayer films were irradiated with 7 MeV X-rays, 10 MeV e-beam, and cobalt-60 gamma at doses between 50 and 70 kGy. Potential polymer changes under these three irradiation modalities were investigated by assessing the general multilayer structural attributes for use in biopharmaceutical applications or in interaction with biopharmaceutical solutions. On the one hand, it was shown that the different irradiation modalities did not significantly affect the thermal properties of the multilayer film constituents. On the other hand, the irradiation modalities did significantly change the quantity of reactive oxygen species generated under irradiation (lower amount of methionine sulfoxide generated by e-beam irradiation than by X-ray or gamma).

This first evaluation of effects of ionizing radiations with this multilayer film reveals that the interaction with EVA/EVOH materials seems to be identical for the high energy photons such as gamma-rays and X-rays. In order to confirm and complete this trend, further studies bracketing and exceeding the established worse case irradiation dose (i.e., ∼59 kGy with gamma-rays) and overlapping the common routine irradiation dose range (i.e., approximately 25–45 kGy) will be undertaken.

## Data Availability

The original contributions presented in the study are included in the article/Supplementary Material, further inquiries can be directed to the corresponding authors.
