# Supplementary material for: Effects of X-Rays, Electron Beam, and Gamma Irradiation on Chemical and Physical Properties of EVA Multilayer Films
Source: Front Chem. 2022 May 11;10:888285. doi: 10.3389/fchem.2022.888285 (PMC9131251; doi:10.3389/fchem.2022.888285)
Supplement: Supplementary file 1 [file DataSheet1.docx]

**Supplementary information -**

**Effects of X-rays, Electron beam and Gamma irradiation on EVA multilayer film properties**

**Nina Girard-Perier^1^, Sylvain R.A. Marque^2^, Nathalie Dupuy^3^, Magalie Claeys-Bruno^3^, Fanny Gaston^1^, Samuel Dorey^1^, Leonard S. Fifield^4^, Yelin Ni^4^, Donghui Li^4^, Witold K. Fuchs^4^, Mark K. Murphy^4^, Suresh D. Pillai^5^, Matt Pharr^5,6^, Larry Nichols^7^**

^1^Sartorius Stedim FMT S.A.S, Z.I. Les Paluds, Avenue de Jouques CS91051, 13781 Aubagne Cedex, France

^2^Aix Marseille Univ, CNRS, ICR, case 551, 13397 Marseille, France

^3^Aix Marseille Univ, Avignon Université, CNRS, IRD, IMBE, Marseille, France

^4^Pacific Northwest National Laboratory, Richland, WA, USA 99354

^5^National Center for Electron Beam Research, Texas A&M University, College Station, TX, USA 77843

^6^Department of Mechanical Engineering, Texas A&M University, College Station, TX, USA 77843

^7^Steri-Tek, Fremont, CA, USA 94538

*Correspondence:

[samuel.dorey@sartorius.com](mailto:samuel.dorey@sartorius.com)

[nathalie.dupuy@univ-amu.fr](mailto:nathalie.dupuy@univ-amu.fr)

[sylvain.marque@univ-amu.fr](mailto:sylvain.marque@univ-amu.fr)

leo.fifield@pnnl.gov

Figure 1 SI. Representative DSC responses of EVA/EVOH/EVA films (series 2) after irradiation under the three modalities up to 60 kGy compared to the unirradiated film. Data were taken from the second heating ramp. Curves were shifted down by a constant as indicated in the figure legend.

Figure 2 SI. Representative FTIR response of EVA/EVOH/EVA films (series 2) after irradiation under the three modalities up to 60 kGy compared to the unirradiated film.

# Experimental section

## High Performance Liquid Chromatography (HPLC)

After the end of each ageing time, bags were filled with a 50 µM solution of methionine in buffer (10 mM NaH­2PO4, 10 mM Na2B4O7•10H2O, 5 mM NaN3, pH 8.2). Sampling was performed after 21 days of storage in the dark, in an air-conditioned room at 20 ± 2°C. The sample was analyzed with an Agilent 1260 HPLC equipped with a quaternary pump (G1311C), an autosampler (G1329B), and a fluorescence detector (G1321B).

Before analysis, the flow rate was set to 3.0 mL/min using vacuum-degassed mobile phases [A, 10 mM NaH­2PO4, 10 mM Na2B4O7•10H2O buffer at pH=8.2; B, acetonitrile:methanol:water (45:45:10, v:v:v) ]. Before use, solvent A was filtered through a 0.22 μm microporous cellulose acetate filtering membrane. As the methionine is non fluorescent, an automated derivatization process is performed to make it fluorescent. The reaction is detailed in the Figure S3. The automated online derivatization (in the autosampler) using an injection program is detailed in **Table 1**. The derivatization reagent used was the ortho-phthaldehyde (OPA).

The gradient program was as follows: 0–13.4 min, 2% B; 13.4 min, 57% B; 13.5-15.8 min, 100% B; 18 min, 2% B. Separation was carried out on an Agilent Poroshell HPH-C18 column (4.6 mm × 100 mm, 2.7 μm particles) used with a pre-column, UHPLC guard Poroshell HPH-C18, 4.6mm. The column was maintained at 40°C +/- 0.8°C in a thermostatted column compartment (G1316A) during the analyses. The fluorescence detector was set to an excitation wavelength of 340 nm and an emission wavelength of 450 nm. The total runtime of the method was 20 min. Chromatographic data were acquired (Figure S4) and evaluated with the HPLC 1260 openlab software. Internal calibration was done by spiking 20 µL of L-Norvaline in each sample and standard. For electron beam and gamma irradiated samples, results are displayed as the average of two batches and the statistical errors represented are calculated on these two batches. For X-ray irradiated samples, the result is displayed for one lot and the statistical error represented is calculated on the seven analyses of the 3.8 µM of methionine sulfoxide standard.

**Table 1: automated online derivatization**

| 1. Draw 2.5 μL from borate vial |
| --- |
| 2. Draw 1.0 μL from sample vial |
| 3. Mix 3.5 μL in wash port five times |
| 4. Wait 0.2 minutes |
| 5. Draw 0.5 μL from OPA vial |
| 6. Mix 4.0 μL in wash port 10 times default speed |
| 7. Mix 4.4 μL in wash port 10 times default speed |
| 8. Draw 32 μL from injection diluent vial (100 mL of mobile phase A and 0.4 mL concentrated H_3_PO_4_) |
| 9. Mix 20 μL in wash port eight times |
| 10. Inject |
| 11. Wait 0.1 minutes |
| 12. Valve bypass |

Figure S3: derivatization reaction of methionine


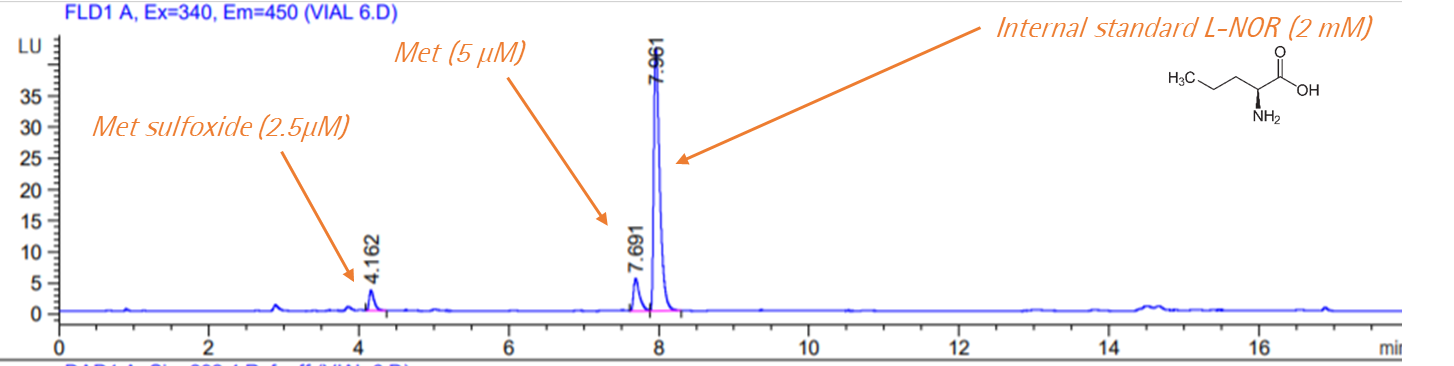


Figure S4: chromatogram of a standard vial

Table 2 SI: Equivalence test outputs with 5°C as equivalence criteria on the EVA material

| Comparison | N | Mean | SD | SE Mean^1^ | Difference | SE | CI 95%^2^ |
| --- | --- | --- | --- | --- | --- | --- | --- |
| 0 vs Gamma 30 kGy | 3 | 85,347 | 0,050332 | 0,029059 | -0,89500 | 0,053671 | (-0,99929; 0) |
|  | 6 | 86,242 | 0,11053 | 0,045123 |  |  |  |
| 0 vs Gamma 45 kGy | 3 | 84,117 | 0,037859 | 0,021858 | -2,1250 | 0,050139 | (-2,22243; 0) |
|  | 6 | 86,242 | 0,11053 | 0,045123 |  |  |  |
| 0 vs Gamma 60 kGy | 3 | 83,397 | 0,19502 | 0,11260 | -2,8450 | 0,12130 | (-3,19920; 0) |
|  | 6 | 86,242 | 0,11053 | 0,045123 |  |  |  |
| 0 vs X-ray 30 kGy | 3 | 84,593 | 0,29704 | 0,17150 | -1,6483 | 0,17733 | (-2,16615; 0) |
|  | 6 | 86,242 | 0,11053 | 0,045123 |  |  |  |
| 0 vs X-ray 45 kGy | 3 | 83,647 | 0,28378 | 0,16384 | -2,5950 | 0,16994 | (-3,09123; 0) |
|  | 6 | 86,242 | 0,11053 | 0,045123 |  |  |  |
| 0 vs X-ray 60 kGy | 3 | 82,937 | 0,38553 | 0,22259 | -3,3050 | 0,22711 | (-3,96817; 0) |
|  | 6 | 86,242 | 0,11053 | 0,045123 |  |  |  |
| 0 vs X-ray 100 kGy | 3 | 83,133 | 0,18009 | 0,10398 | -3,1083 | 0,11335 | (-3,43930; 0) |
|  | 6 | 86,242 | 0,11053 | 0,045123 |  |  |  |
| 0 vs Ebeam 30 kGy | 3 | 84,263 | 0,090738 | 0,052387 | -1,9783 | 0,069142 | (-2,12573; 0) |
|  | 6 | 86,242 | 0,11053 | 0,045123 |  |  |  |
| 0 vs Ebeam 45 kGy | 3 | 83,823 | 0,050332 | 0,029059 | -2,4183 | 0,053671 | (-2,52263; 0) |
|  | 6 | 86,242 | 0,11053 | 0,045123 |  |  |  |
| 0 vs Ebeam 60 kGy | 3 | 83,073 | 0,050332 | 0,029059 | -3,1683 | 0,053671 | (-3,27263; 0) |
|  | 6 | 86,242 | 0,11053 | 0,045123 |  |  |  |

1 SE mean (Standard error of the mean), 2 Confidence interval (CI) at 95%

Table 2 SI: Equivalence test outputs with 5°C as equivalence criteria on the PE material

| Comparison | N | Mean | SD | SE Mean^1^ | Difference | SE | CI 95%^2^ |
| --- | --- | --- | --- | --- | --- | --- | --- |
| 0 vs Gamma 30 kGy | 3 | 108,13 | 0,030551 | 0,017638 | -0,49833 | 0,11326 | (-0,726552; 0) |
|  | 6 | 108,63 | 0,27404 | 0,11188 |  |  |  |
| 0 vs Gamma 45 kGy | 3 | 107,96 | 0,28160 | 0,16258 | -0,67167 | 0,19736 | (-1,13612; 0) |
|  | 6 | 108,63 | 0,27404 | 0,11188 |  |  |  |
| 0 vs Gamma 60 kGy | 3 | 107,61 | 0,25027 | 0,14449 | -1,0250 | 0,18274 | (-1,41457; 0) |
|  | 6 | 108,63 | 0,27404 | 0,11188 |  |  |  |
| 0 vs X-ray 30 kGy | 3 | 108,17 | 0,10970 | 0,063333 | -0,46500 | 0,12856 | (-0,714812; 0) |
|  | 6 | 108,63 | 0,27404 | 0,11188 |  |  |  |
| 0 vs X-ray 45 kGy | 3 | 107,91 | 0,30827 | 0,17798 | -0,71833 | 0,21022 | (-1,21307; 0) |
|  | 6 | 108,63 | 0,27404 | 0,11188 |  |  |  |
| 0 vs X-ray 60 kGy | 3 | 107,53 | 0,24269 | 0,14012 | -1,1017 | 0,17930 | (-1,48391; 0) |
|  | 6 | 108,63 | 0,27404 | 0,11188 |  |  |  |
| 0 vs X-ray 100 kGy | 3 | 107,53 | 0,19140 | 0,11050 | -1,1050 | 0,15725 | (-1,42186; 0) |
|  | 6 | 108,63 | 0,27404 | 0,11188 |  |  |  |
| 0 vs Ebeam 30 kGy | 3 | 108,16 | 0,095394 | 0,055076 | -0,47167 | 0,12470 | (-0,713976; 0) |
|  | 6 | 108,63 | 0,27404 | 0,11188 |  |  |  |
| 0 vs Ebeam 45 kGy | 3 | 107,95 | 0,011547 | 0,0066667 | -0,68500 | 0,11207 | (-0,910834; 0) |
|  | 6 | 108,63 | 0,27404 | 0,11188 |  |  |  |
| 0 vs Ebeam 60 kGy | 3 | 107,45 | 0,26502 | 0,15301 | -1,1850 | 0,18954 | (-1,58908; 0) |
|  | 6 | 108,63 | 0,27404 | 0,11188 |  |  |  |

1 SE mean (Standard error of the mean), 2 Confidence interval (CI) at 95%

Table 3 SI: Equivalence test outputs with 5°C as equivalence criteria on the EVOH material

| **Comparison** | **N** | **Mean** | **SD** | **SE Mean^1^** | **Difference** | **SE** | **CI 95%^2^** |
| --- | --- | --- | --- | --- | --- | --- | --- |
| 0 vs Gamma 30 kGy | 3 | 159,30 | 0,10017 | 0,057831 | -4,7350 | 0,092919 | (-4,91556; 0) |
|  | 6 | 164,03 | 0,17815 | 0,072729 |  |  |  |
| 0 vs Gamma 45 kGy | 3 | 159,36 | 0,036056 | 0,020817 | -4,6717 | 0,075649 | (-4,82410; 0) |
|  | 6 | 164,03 | 0,17815 | 0,072729 |  |  |  |
| 0 vs Gamma 60 kGy | 3 | 159,81 | 0,38371 | 0,22154 | -4,2250 | 0,23317 | (-4,90585; 0) |
|  | 6 | 164,03 | 0,17815 | 0,072729 |  |  |  |
| 0 vs X-ray 30 kGy | 3 | 161,13 | 0,060000 | 0,034641 | -2,9017 | 0,080557 | (-3,05820; 0) |
|  | 6 | 164,03 | 0,17815 | 0,072729 |  |  |  |
| 0 vs X-ray 45 kGy | 3 | 161,10 | 0,030551 | 0,017638 | -2,9283 | 0,074837 | (-3,07913; 0) |
|  | 6 | 164,03 | 0,17815 | 0,072729 |  |  |  |
| 0 vs X-ray 60 kGy | 3 | 160,85 | 0,25146 | 0,14518 | -3,1783 | 0,16238 | (-3,56047; 0) |
|  | 6 | 164,03 | 0,17815 | 0,072729 |  |  |  |
| 0 vs X-ray 100 kGy | 3 | 158,84 | 0,10017 | 0,057831 | -5,1883 | 0,092919 | (-5,36889; 0) |
|  | 6 | 164,03 | 0,17815 | 0,072729 |  |  |  |
| 0 vs Ebeam 30 kGy | 3 | 161,81 | 0,34847 | 0,20119 | -2,2250 | 0,21393 | (-2,84968; 0) |
|  | 6 | 164,03 | 0,17815 | 0,072729 |  |  |  |
| 0 vs Ebeam 45 kGy | 3 | 160,45 | 0,13577 | 0,078387 | -3,5783 | 0,10693 | (-3,79380; 0) |
|  | 6 | 164,03 | 0,17815 | 0,072729 |  |  |  |
| 0 vs Ebeam 60 kGy | 3 | 160,45 | 0,13577 | 0,078387 | -3,5783 | 0,10693 | (-3,79380; 0) |
|  | 6 | 164,03 | 0,17815 | 0,072729 |  |  |  |
| Gamma 30 kGy vs X-ray 30 kGy | 3 | 161,13 | 0,060000 | 0,034641 | 1,8333 | 0,067412 | (0; 1,99198) |
|  | 3 | 159,30 | 0,10017 | 0,057831 |  |  |  |
| Gamma 30 kGy vs X-ray 45 kGy | 3 | 161,10 | 0,030551 | 0,017638 | 1,8067 | 0,060461 | (0; 1,98321) |
|  | 3 | 159,30 | 0,10017 | 0,057831 |  |  |  |
| Gamma 30 kGy vs X-ray 60 kGy | 3 | 160,85 | 0,25146 | 0,14518 | 1,5567 | 0,15628 | (0; 2,01299) |
|  | 3 | 159,30 | 0,10017 | 0,057831 |  |  |  |
| Gamma 30 kGy vs X-ray 100 kGy | 3 | 158,84 | 0,10017 | 0,057831 | -0,45333 | 0,081786 | (-0,645805; 0) |
|  | 3 | 159,30 | 0,10017 | 0,057831 |  |  |  |
| Gamma 45kGy vs X-ray 100 kGy | 3 | 158,84 | 0,10017 | 0,057831 | -0,51667 | 0,061464 | (-0,696140; 0) |
|  | 3 | 159,36 | 0,036056 | 0,020817 |  |  |  |
| Gamma 60 kGy vs X-ray 100 kGy | 3 | 158,84 | 0,10017 | 0,057831 | -0,96333 | 0,22896 | (-1,63189; 0) |
|  | 3 | 159,81 | 0,38371 | 0,22154 |  |  |  |

1 SE mean (Standard error of the mean), 2 Confidence interval (CI) at 95%
